# Evaluation of Cardiac Biomarkers in Lambs with White Muscle Disease

**DOI:** 10.3390/vetsci12080774

**Published:** 2025-08-19

**Authors:** Gencay Ekinci, Murat Eren, Kübra Yağlı, Celil Bendeş, Görkem Ekebaş, Emre Tüfekçi, Sefa Güzel, Latife Çakır Bayram, Ali Cesur Onmaz, Vehbi Güneş, Mehmet Çitil, İhsan Keleş

**Affiliations:** 1 Department of Internal Medicine, Faculty of Veterinary Medicine, Erciyes University, Kayseri 38280, Türkiye; aonmaz@erciyes.edu.tr (A.C.O.); vgunes@erciyes.edu.tr (V.G.); mehmetcitil@erciyes.edu.tr (M.Ç.); ihsankeles@erciyes.edu.tr (İ.K.); 2 Department of Animal Nutrition and Nutritional Diseases, Institute of Health Sciences, Erciyes University, Kayseri 38280, Türkiye; murateren@tarimorman.gov.tr; 3 Department of Pathology, Faculty of Veterinary Medicine, Erciyes University, Kayseri 38280, Türkiye; kubrayagli@erciyes.edu.tr (K.Y.); gekebas@erciyes.edu.tr (G.E.); lcakir@erciyes.edu.tr (L.Ç.B.); 4 Department of Veterinary Internal Medicine, Institute of Health Sciences, Erciyes University, Kayseri 38280, Türkiye; celilbendes@erciyes.edu.tr (C.B.); sefa.guzel@tarimorman.gov.tr (S.G.); 5 Department of Wild Animal Diseases, Faculty of Veterinary Medicine, Erciyes University, Kayseri 38280, Türkiye; emretufekci@erciyes.edu.tr

**Keywords:** cardiac biomarkers, galectin-3, lamb, N-terminal pro-brain natriuretic peptide, selenium, white muscle disease

## Abstract

White muscle disease (WMD) is a degenerative condition of the skeletal and/or cardiac muscle associated with selenium (Se) and/or vitamin E deficiency, which can present in acute, subacute, or chronic forms, and is most commonly observed in young, rapidly growing animals, though it may also occur in older individuals. This study aims to determine the serum concentrations of galectin-3 (Gal-3), cardiac troponin I (cTnI), and N-terminal pro-brain natriuretic peptide (NT-proBNP), as well as the activity of creatine kinase-myocardial band (CK-MB), in lambs diagnosed with WMD, and to investigate the diagnostic potential of these biomarkers in the evaluation of myocardial injury and skeletal and/or cardiac muscle necrosis associated with WMD. A total of 50 lambs, 20 healthy and 30 with WMD, were included in the study. The diagnosis of WMD was made based on clinical signs, laboratory results, necropsy findings, and blood vitamin E and Se concentrations. The lambs in the WMD group were categorized into two subgroups: confirmed, severe aWMD (acute animals, *n* = 10) lambs and presumed sWMD (subacute animals, *n* = 20), based on the clinical progression and severity of the disease. Serum levels of NT-proBNP, Gal-3, and cTnI were assessed using the ELISA technique. Levels of cTnI and CK-MB indicative of myocardial injury were found to be considerably elevated in the aWMD group (*p* < 0.001) in comparison to both the sWMD and control groups. CK-MB showed a strong positive correlation with cTnI (r = 0.819, *p* < 0.001). The serum concentrations of Gal-3 and NT-proBNP in healthy lambs were 2.55 ± 0.52 ng/mL and 3.28 ± 0.71 ng/mL, respectively. Serum Gal-3 concentrations were measured as 2.99 ± 0.44 ng/mL in the aWMD group and 3.07 ± 0.42 ng/mL in the sWMD group, while NT-proBNP concentrations were 2.15 ± 0.32 ng/mL and 2.64 ± 0.55 ng/mL in the aWMD and sWMD groups, respectively. No statistically significant differences were found in serum Gal-3 or NT-proBNP levels among the three groups (*p* > 0.05). In conclusion, this study is the first investigation assessing serum concentrations of Gal-3 and NT-proBNP in lambs afflicted with WMD. The results suggest that Gal-3 and NT-proBNP are ineffective biomarkers for assessing myocardial injury and skeletal and/or cardiac muscle necrosis associated with WMD in lambs. However, cTnI and CK-MB appear to be significant indicators of cardiac involvement in both acute and subacute scenarios. Further research is required to elucidate the molecular function of Gal-3 in muscle and cardiac disease in lambs afflicted with WMD.

## Simple Summary

White muscle disease (WMD) is a degenerative condition of the skeletal and/or cardiac muscle associated with selenium (Se) and/or vitamin E deficiency. This study aims to determine the serum concentrations of galectin-3 (Gal-3), cardiac troponin I (cTnI), and N-terminal pro-brain natriuretic peptide (NT-proBNP), as well as the activity of creatine kinase-myocardial band (CK-MB), in lambs diagnosed with WMD, and to investigate the diagnostic potential of these biomarkers in the evaluation of myocardial injury and skeletal and/or cardiac muscle necrosis associated with WMD. Levels of cTnI and CK-MB indicative of myocardial injury were found to be considerably elevated in the aWMD group (*p* < 0.001) in comparison to both the sWMD and control groups. CK-MB showed a strong positive correlation with cTnI (r = 0.819, *p* < 0.001). The serum concentrations of Gal-3 and NT-proBNP in healthy lambs were 3.28 ± 0.71 ng/mL and 2.55 ± 0.52 ng/mL, respectively. Serum Gal-3 concentrations were measured as 2.99 ± 0.44 ng/mL in the aWMD group and 3.07 ± 0.42 ng/mL in the sWMD group, while NT-proBNP concentrations were 2.15 ± 0.32 ng/mL and 2.64 ± 0.55 ng/mL in the aWMD and sWMD groups, respectively. No statistically significant differences were found in serum Gal-3 or NT-proBNP levels among the three groups (*p* > 0.05).

## 1. Introduction

White muscle disease (WMD), also known as nutritional muscular dystrophy, is a degenerative condition of the skeletal and/or cardiac muscle associated with selenium (Se) and/or vitamin E deficiency, which can present in acute, subacute, or chronic forms, and is most commonly observed in young, rapidly growing animals, though it may also occur in older individuals [1,2]. Providing ewes with selenium-deficient diets during gestation elevates the likelihood of WMD occurrence in lambs [3,4]. Multiple environmental and metabolic factors, including physical activity, excessive caloric intake, elevated consumption of unsaturated fatty acids, copper deficiency, and stress significantly contribute to the disease’s progression [4,5]. The acute variant of this disease, predominantly observed in lambs and infrequently in calves, is marked by abrupt mortality, whereas the subacute or chronic variant is distinguished by lethargy, muscular debilitation, locomotor dysfunction, and stunted growth [4,5].

Oxidative stress significantly contributes to the etiology of WMD [6]. Se and vitamin E are the primary constituents of the antioxidant defense system. Reactive oxygen species (ROS) increase due to inadequacies, resulting in oxidative injury to muscle cells. The impairment of cell membranes facilitates the influx of extracellular calcium into the cell, thereby activating calcium-dependent proteases [4]. These enzymes decompose myofibrils and other cellular components, resulting in degeneration and necrosis of muscle tissue [7]. The disease primarily affects skeletal muscle tissue; however, in a significant proportion of cases, cardiac muscle tissue may also be involved. In response to the muscle injury, the animal mounts inflammatory and fibrotic reactions within the affected musculature [4]. Throughout this process, degeneration, necrosis, fibrosis, and calcification may manifest in the cardiac muscle, leading to respiratory difficulty, arrhythmia, and sudden death [8].

N-terminal pro-brain natriuretic peptide (NT-proBNP) is the inactive amino-terminal portion of brain natriuretic peptide (BNP), secreted by cardiac muscle cells into the bloodstream via ventricular myocytes in response to heart wall stress [9]. Blood levels rise markedly, particularly in circumstances like congestive heart failure, characterized by cardiac stress and volume overload [10]. In human and small animal medicine, NT-proBNP is extensively utilized for the diagnosis, prognosis, and treatment monitoring of conditions including congestive heart failure, left ventricular dysfunction, myocardial infarction, and hypertrophic cardiomyopathy [9,11,12,13,14]. Assessing NT-proBNP concentrations in ruminants is seen as a promising method for diagnosing and monitoring heart disorders [15,16]. Nonetheless, due to the restricted research in this domain, routine use in clinical practice has not yet been realized.

Galectin-3 (Gal-3), a β-galactoside-binding lectin, is pivotal in inflammation, fibrosis, and cellular repair mechanisms [17]. Gal-3 facilitates the onset and enhancement of the acute inflammatory response by recruiting macrophages to damage sites. It perpetuates the chronic inflammatory condition by the activation of pro-inflammatory pathways [17,18,19]. Gal-3 is a crucial mediator in the pathophysiology of lung-related inflammatory and fibrotic disorders, including idiopathic pulmonary fibrosis (IPF) and COVID-19 [20]. Gal-3 expression is elevated in fibrotic kidney specimens [21]. Recent investigations have identified Gal-3 as a novel biomarker for cardiovascular illnesses, potentially serving as a significant predictor of disease progression in patients with heart failure [17,19,22,23].

In lambs with WMD, the right and left ventricles and the interventricular septum may be affected, although the exact prevalence of cardiac involvement remains unclear [8]. Histologically, muscle fibers show hypercontraction, fragmentation, mineralization, and macrophage infiltration [24]. Cardiac degeneration, necrosis, calcification, and fibrosis are characteristic features [8]. These structural damages lead to increased serum levels of cardiac biomarkers such as creatine kinase-myocardial band (CK-MB) and cardiac troponin I (cTnI), which indicate acute myocardial injury [4,25]. WMD impairs cardiac contractility and conduction, often causing arrhythmias or heart failure, which may result in sudden death [4]. Recently, NT-proBNP and Gal-3 have emerged as noninvasive biomarkers for heart failure and fibrosis in humans and small animals [17,23]. There is not yet sufficient data in the literature regarding the normal serum levels of Gal-3 and NT-proBNP in lambs and changes in disease states. However, determination of serum levels of these biomarkers may provide important information in determining cardiac dysfunction or heart failure caused by WMD. Further studies in this field will contribute to a better understanding of the clinical value of Gal-3 and NT-proBNP in lambs.

This study aims to determine the serum concentrations of Gal-3, cTnI and NT-proBNP, as well as the activity of CK-MB, in lambs diagnosed with WMD, and to investigate the diagnostic potential of these biomarkers in the evaluation of myocardial injury and skeletal and/or cardiac muscle necrosis associated with WMD.

## 2. Materials and Methods

### 2.1. Study Design

Prospective, case-control study.

### 2.2. Ethics Statement

This study was approved by the Erciyes University Animal Experiments Local Ethics Committee (ERU-HADYEK) (No: 23/211).

### 2.3. Animals

A total of 50 lambs, 20 healthy and 30 with a WMD diagnosis, were included in the study. The study was conducted between 2023 and 2024. A purposive sampling method was used in the sampling process. The sample size was determined using G*Power 3.1 software [26] in order to achieve sufficient power in comparative statistical analyses. Power analysis was performed assuming a significance level of α = 0.05, a statistical power of 0.80 (1-β) and a medium effect size (Cohen’s d = 0.5); it was calculated that at least 42 lambs should be included in the study. Accordingly, the number of samples was increased, and a total of 50 lambs were included in the study.

The lambs in the WMD group were categorized into two subgroups: confirmed, severe aWMD (acute animals, *n* = 10) lambs and presumed sWMD (subacute animals, *n* = 20), based on the clinical progression and severity of the disease.

The lambs in the aWMD group (*n* = 10) were transported from various traditional sheep farms (*n* = 10) in Kayseri and surrounding provinces to Erciyes University Faculty of Veterinary Medicine, Veterinary Teaching Hospital (ERU-VTH), between 2023 and 2024. These lambs were suspected of having WMD based on clinical examination and were brought in for necropsy evaluation. The definitive diagnosis of the disease was confirmed post-mortem examination following euthanasia. The diagnosis of aWMD was confirmed based on clinical evaluation (day 1), serum biochemical parameters (LDH, CK, AST, and CK-MB enzyme activities), vitamin E and selenium (Se) levels, and histopathological examinations. The necropsy examination of the lambs in the aWMD group revealed characteristic indications of both skeletal and cardiac muscle involvement in the disease. The clinical signs in this group were severe, including marked muscle weakness, difficulty in movement, dyspnea, and tachycardia (>160 bpm). These severe clinical signs reflected the acute and rapidly progressing nature of the disease in the confirmed, severe WMD (acute animals) lambs.

The sWMD group (*n* = 20) comprised Akkaraman breed lambs, aged 4–8 weeks, of both sexes, sourced in 2023 from a sheep farm in Yeşilhisar village, Boğazlıyan district, Yozgat province (Türkiye). Two of these lambs were suspected of having WMD based on anamnesis, clinical examination, biochemical tests (including LDH, CK, AST, and CK-MB enzyme activities), and vitamin E and selenium (Se) levels. These two lambs were subsequently euthanized and referred to Erciyes University Faculty of Veterinary Medicine, Veterinary Teaching Hospital (ERU-VTH) for necropsy and histopathological evaluation. Histopathological examination of these two lambs revealed typical lesions characteristic of skeletal muscle involvement. Three days later, blood samples were collected from other lambs on the same farm exhibiting similar clinical signs. Lambs exhibiting typical clinical signs of WMD, elevated serum enzyme activities (LDH, CK, AST, and CK-MB), and decreased vitamin E and Se concentrations were considered to have the subacute form of the disease. The most notable clinical signs in this group included a stiff and uncoordinated gait, short and rigid steps, lameness, and dyspnea. These signs were generally milder compared to the acute form and were assumed to represent presumed WMD (subacute animals). However, muscle biopsy or histopathological examination was not performed on these lambs, except for the two lambs brought to the Veterinary Teaching Hospital (VTH).

Following clinical examination and sample collection, lambs in the sWMD group (*n* = 18) received supportive treatment consisting of vitamin E + Se (Selephos^®^, Topkim, Türkiye) and cobalamin + phosphorus (Catasol^®^ 10%, Bayer, Germany). Specifically, vitamin E + Se was administered intramuscularly at a dose of 1 mL per 10 kg body weights, containing vitamin E (30 mg/mL) and Se (0.6 mg/mL), as a single dose. A second vitamin E + Se injection was administered to lambs that did not show complete clinical improvement within two days following the initial dose. Additionally, cyanocobalamin and phosphorus were administered intramuscularly at a dose of 1 mL per 10 kg body weight, once daily for three consecutive days. Lambs in this group were followed for 28 days. All lambs (*n* = 18) in the sWMD group showed a favorable response to the treatment, except for the two lambs that were euthanized.

The Akkaraman lambs in the control group (*n* = 20) were selected from a similar age group (4–8 weeks), did not show any clinical signs of WMD, and were within normal reference ranges in terms of hematology, biochemical assays, selenium and vitamin E. No injection or supplementation was administered to the lambs in the control group. This group represents the natural selenium and vitamin E status under field conditions.

### 2.4. Criteria for Inclusion and Exclusion

Lambs were included in the study if they exhibited clinical signs consistent with WMD, such as a stiff and uncoordinated gait, short and rigid steps, general weakness, dyspnea, tachycardia, reluctance to move, and difficulty standing. These signs were used to identify lambs suspected of having WMD prior to further diagnostic confirmation; those with diminished selenium and/or vitamin E levels; those with elevated LDH, CK, AST, and CK-MB enzyme activities in biochemical assessments; those displaying both macroscopic and microscopic pathological lesions in cardiac/skeletal muscles during necropsy; and those with negative PCR results for foot and mouth disease were incorporated into the study. The exclusion criteria encompassed lambs with congenital myopathies, myositis resulting from bacterial infections (*Clostridium* spp.), and electrolyte imbalances such as hypoglycemia and hypokalemia that could induce muscular weakening and degeneration.

### 2.5. Clinical Assessment

The lambs in the study underwent a comprehensive physical examination, which encompassed body temperature (°C), heart rate (bpm), respiration rate (breaths/min), and an evaluation of their general condition.

### 2.6. Blood Sampling

For complete blood count analyses, 2 mL blood samples were taken from the jugular vein via puncture into blood collection tubes containing K_2_ EDTA (BD Vacutainer^®^, Becton Dickinson, Franklin Lakes, NJ, USA (NA)). In addition, 5 mL blood samples were taken from the jugular vein via puncture into yellow-capped (gel) blood collection tubes (BD Vacutainer^®^, Becton Dickinson, Franklin Lakes, NJ, USA (NA)) for serum collection. Blood samples were centrifuged at 3000× *g* for 15 min and after that stored in 0.5 mL aliquots at −20 °C under appropriate storage conditions.

### 2.7. Selenium and Vitamin E Analysis

Selenium analyses from serum samples were performed for all animals in each group (aWMD, *n* = 10; sWMD, *n* = 20; Control, *n* = 20) by Erciyes University Technology Research and Application Center (Kayseri, Türkiye) through service procurement. An inductively coupled plasma-mass spectrometry device (Agilent 7500a, Agilent Technologies, Santa Clara, CA, USA (NA)) was used in the analysis. Vitamin E concentrations were measured in serum samples from all animals (aWMD, *n* = 10; sWMD, *n* = 20; Control, *n* = 20) using the high-performance liquid chromatography (HPLC) method, employing a Chromsystems^®^ HPLC column specifically designed for fat-soluble vitamins. The analyses were conducted on a Shimadzu LC-20AT HPLC system (Shimadzu Corporation, Kyoto, Japan).

### 2.8. Complete Blood Count Analysis

Hematological analyses (aWMD, *n* = 10; sWMD, *n* = 20; Control, *n* = 20) were performed with a complete blood count device (Exigo EosVet, Boule Medical AB, Stockholm, Sweden).

### 2.9. Analysis of Biochemical Parameters and Glutathione Peroxidase Activity

Analyses of creatine kinase (CK), creatine kinase myocardial band (CK-MB), lactate dehydrogenase (LDH), and aspartate transaminase (AST) were conducted at the Clinical Biochemistry and Hematology Laboratory of Erciyes University Faculty of Veterinary Medicine (aWMD, *n* = 10; sWMD, *n* = 20; Control, *n* = 20). Analyses of glutathione peroxidase (GSH-Px) were conducted utilizing a commercial ELISA kit. Serum samples were examined using an ELISA kit for sheep (QS00045Sp, Sunlong Biotech Co., Ltd., Hangzhou, China (AS)) following the manufacturer’s procedure.

### 2.10. Analyses of NT-Probnp, cTnI, and Galectin-3

Serum samples were examined utilizing the sheep NT-proBNP ELISA Kit (SL00151SP, Sunlong Biotech Co., Ltd., Hangzhou, China (AS)), sheep cTnI ELISA Kit (SL00150SP, Sunlong Biotech Co., Ltd., Hangzhou, China (AS)), and sheep Galectin-3 ELISA Kit (SL00153SP, Sunlong Biotech Co., Ltd., Hangzhou, China (AS)) following the manufacturer’s instructions (aWMD, *n* = 10; sWMD, *n* = 20; Control, *n* = 20). Standards were analyzed in duplicate, and absorbance was measured at 450 nm using a microplate reader (BioTek ELX800, Absorbance Microplate Reader, BioTek Instruments, Colmar, France).

### 2.11. Histopathological Examination

In this study, skeletal and cardiac muscle samples were collected from all lambs in the aWMD group (*n* = 10) and from two lambs in the sWMD group for histopathological examination. Samples included intercostal (*Musculi intercostales interni et externi*), forelimb muscles (*M. biceps brachii*, *M. triceps brachii*), and gluteal (*M. semitendinosus*, *M. semimembranosus*) muscles, as well as sections from the epicardium, endocardium, and ventricular myocardium. Tissue samples for histopathological examination were preserved in 10% neutral buffered formalin. Tissue samples underwent dehydration using graded ethanol concentrations before being processed automatically with the Leica TP1020 Semi-Closed Benchtop Tissue Processor and subsequently embedded in paraffin blocks. Deparaffinized slices were stained with Hematoxylin-Eosin (HE) using the HM 340E Electronic Rotary Microtome from Thermo Scientific (Waltham, WA, USA). All preparations were documented through photography (minimum of four fields per preparation, utilizing ×10, ×40, and ×100 lenses) with a (BX-51, Olympus, Tokyo, Japan (AS)) equipped with a digital camera (Olympus DP71, Olympus Corporation, Tokyo, Japan (AS)) and digital software (DP Controller and DP Manager, Olympus Corporation, Tokyo, Japan (AS)).

### 2.12. Statistical Analyses

Statistical analyses were conducted utilizing the SPSS 25.0 software (IBM Corp., Chicago, IL, USA (NA)). Descriptive statistics are displayed as frequencies and percentages. The normality of the data distribution was evaluated utilizing the Shapiro–Wilk test in conjunction with histograms and Q-Q plots. The results were presented as mean ± standard deviation for normally distributed data and as median (1st and 3rd quartile) for non-normally distributed data. Group comparisons were conducted using the ANOVA test, where parametric assumptions were satisfied, and the Kruskal–Wallis test when they were not. The Spearman’s rho test was employed for correlation analysis. The Bonferroni and Tukey HSD tests were used in post hoc comparisons. ROC analysis was conducted to evaluate diagnostic performance, and the optimal cut-off point was established using the Youden Index. The graphs were generated utilizing GraphPad Prism 9.0 (GraphPad Software, Inc., San Diego, CA, USA). A *p*-value less than 0.05 was considered statistically significant.

## 3. Results

Groups were roughly balanced in terms of sex (aWMD group 40% male, 60% female; sWMD group 45% male and 55% female, control group 50% male, 50% female). Ages of the aWMD group ranged from 15 to 52 days, the sWMD group from 24 to 54 days, and the control group from 20 to 56 days. Mean body weights were 7.70 ± 1.79 kg (range 4–10 kg), 12.60 ± 2.06 kg (range 7–17 kg), and 10.26 ± 1.79 kg (range 8–16 kg) for aWMD, sWMD, and control groups, respectively.

Significant differences were observed among the groups in terms of body temperature, heart rate, and respiratory rate. The aWMD group showed the highest values for all parameters, with significantly elevated body temperature (39.7 °C), heart rate (235.0 beats/min), and respiratory rate (95.4 breaths/min) compared to the sWMD and control groups (*p* < 0.05) (Table 1).

The clinical signs commonly observed in the lambs in the aWMD group included dyspnea (labored and rapid breathing), tachycardia, weak peripheral pulse, cold extremities, persistent recumbency, weakness, and loss of appetite. Some lambs were found in lateral or sternal recumbency and showed signs consistent with an agonal state. WMD-specific lesions in the heart, skeletal muscles, and diaphragm were confirmed by necropsy examination in lambs included in the aWMD group.

In the sWMD group, clinical manifestations were less severe. Prominent clinical indicators included reluctance to ambulate, dyspnea (labored and rapid breathing), weakness, trouble in standing, kyphosis, lameness, and a rigid gait characterized by upright, short steps. In some cases, there was growth retardation, inadequate muscle development, muscle rigidity, and tremors. In the sWMD group of lambs, clinical signs subsided and marked improvement was observed by day 28 following treatment with vitamin E, selenium, cobalamin, and phosphorus. Cobalamin and phosphorus were included to support energy metabolism, red blood cell production, and muscle function, complementing the antioxidant effects of vitamin E and selenium.

### 3.1. Macroscopic Findings

**Figure 1:**
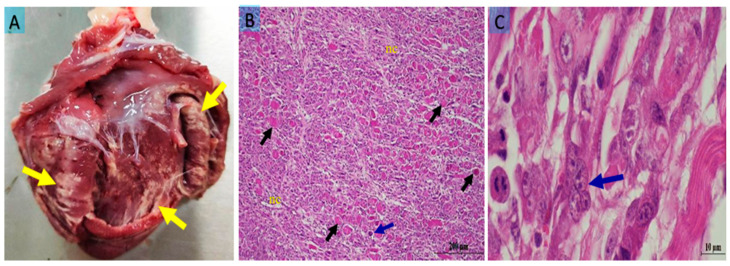
(**A**) Pale yellowish-white necrotic muscle fibers in the heart (yellow arrows); (**B**) hyaline degeneration (black arrows), Zenker’s necrosis (nc), and a multinucleated cell indicating myocyte regeneration (blue arrow) in the myocardium; (**C**) a multinucleated giant cell (blue arrow) in the myocardium at high magnification. Bar: 200 μm (**A**); 10 μm (**B**). Staining: hematoxylin x eosin (**A**,**B**).

**Figure 2:**
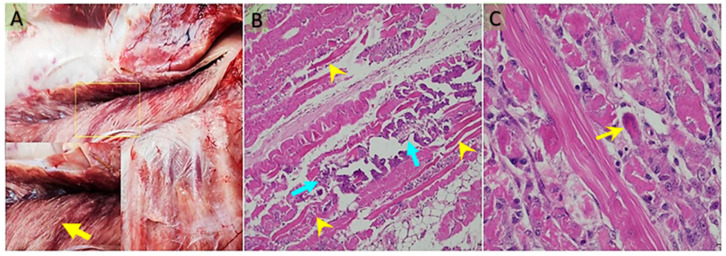
(**A**) Pale, whitish necrotic muscle fibers in the longissimus muscle (yellow arrow) (the boxed area is shown at higher magnification). (**B**) In the longissimus dorsi muscle, hyaline degeneration and Zenker’s-type necrosis (blue arrow), along with areas of dystrophic calcification (yellow arrowhead). (**C**) In the subepicardial regions of the myocardium, prominent hyaline degeneration and Zenker’s-type necrosis are observed, accompanied by bizarre, multinucleated giant cells of presumed myocytic origin, likely associated with a regenerative response (yellow arrow). Bar: 200 μm (**A**); 50 μm (**B**). Stain: hematoxylin x eosin (**A**,**B**).

Costal muscles (*M. intercostales interni*, *M. intercostales externi*), forelimb muscles (*M. biceps brachii*, *M. triceps brachii*), and gluteal muscles (*M. semitendinosus*, *M. semimembranosus*) were examined. In some cases, all skeletal muscles were affected, but in most cases, the lesions were prominent in the costal and gluteal regions. In the heart, pale white foci were observed in the epicardium, endocardium, and ventricles (Figure 1A). The muscles showed pale, grayish-white necrotic and calcified areas (Figure 2A). No macroscopic abnormalities were detected in other organs examined.

### 3.2. Microscopic Findings

Similar histopathological changes were observed in both the heart and gluteal muscles. Muscle fibers exhibited disruption of their normal architecture and appeared swollen. In transverse sections, nuclei were pyknotic. Longitudinal sections showed loss of cross-striations, swelling, homogeneous eosinophilic cytoplasm, and pyknotic nuclei. Additionally, areas of dystrophic calcification were detected in these regions.

The sarcoplasm of the affected myofibers was filled with homogeneous eosinophilic material, with obliteration of the normal cross-striations. Necrotic regions exhibited edema, increased cellularity, and infiltration of degenerating fibers by macrophages.

In the myocardium, degeneration of myofibrils, necrosis of both cardiac myocytes and endocardial cells, sarcolemmal separation, and loss of striation were noted. Hyaline degeneration, coagulative necrosis, and Zenker’s necrosis of the myofibrils were prominent (Figure 1B), along with infiltration of mononuclear inflammatory cells. Multinucleated giant cells were observed surrounding regenerating myofibrils, particularly in the subepicardial regions (Figure 1C).

Histopathological examination of the skeletal muscles revealed dystrophic calcification around necrotic and degenerated myofibers, hyaline degeneration, and Zenker’s necrosis within dystrophic myofibrils (Figure 2B). Mononuclear cell infiltration and irregular multinucleated giant cells were also observed around degenerated muscle fibers (Figure 2C).

### 3.3. Selenium and Vitamin E Levels

The Se concentration in the serum of lambs in the case group (aWMD: 18.31 ± 6.12 ng/mL and sWMD: 43.06 ± 5.01 ng/mL) was found to be significantly lower than that of the control group (133.31 ± 34.85 ng/mL) (*p* = 0.001, *p* = 0.006, respectively).

The concentration of vitamin E in the serum of lambs in the case group (aWMD: 0.15 mg/L and sWMD: 0.25 mg/L) was significantly lower than that of the control group (1.97 mg/L) (*p* < 0.001).

### 3.4. Complete Blood Count Findings

**Figure A1:**
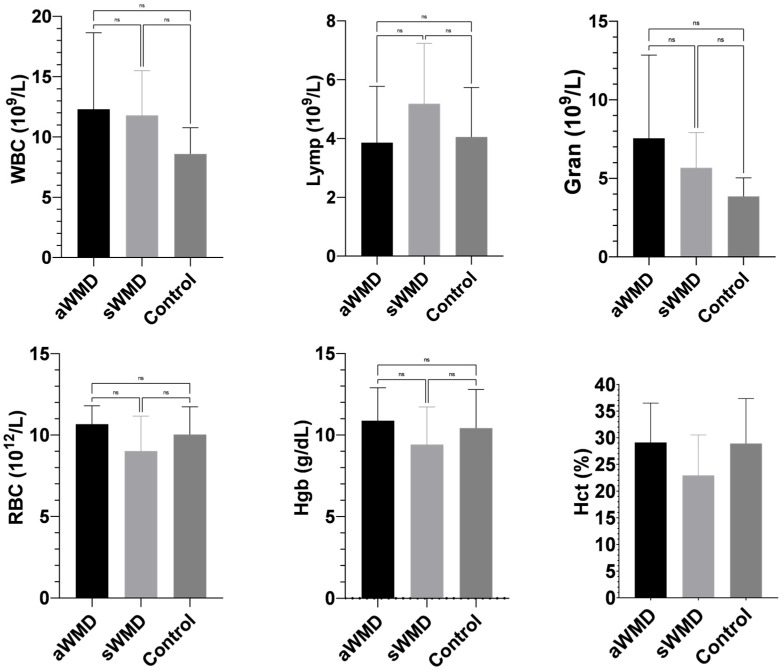
Comparison of complete blood count parameters between case and control groups. aWMD: confirmed, severe white muscle disease (acute animals); sWMD: presumed white muscle disease (subacute animals). Control: healthy lambs. RBC: red blood cell; Hct: hematocrit; Hgb: hemoglobin concentration; Lymph: lymphocyte; Gran: granulocyte; WBC: white blood cell. ns: non-significant.

The case (aWMD, sWMD) and control groups showed similar results in terms of complete blood count parameters, and the difference between them was not found to be statistically significant (*p* > 0.05) (Appendix B; Figure A1).

### 3.5. Biochemical Findings

**Figure 3:**
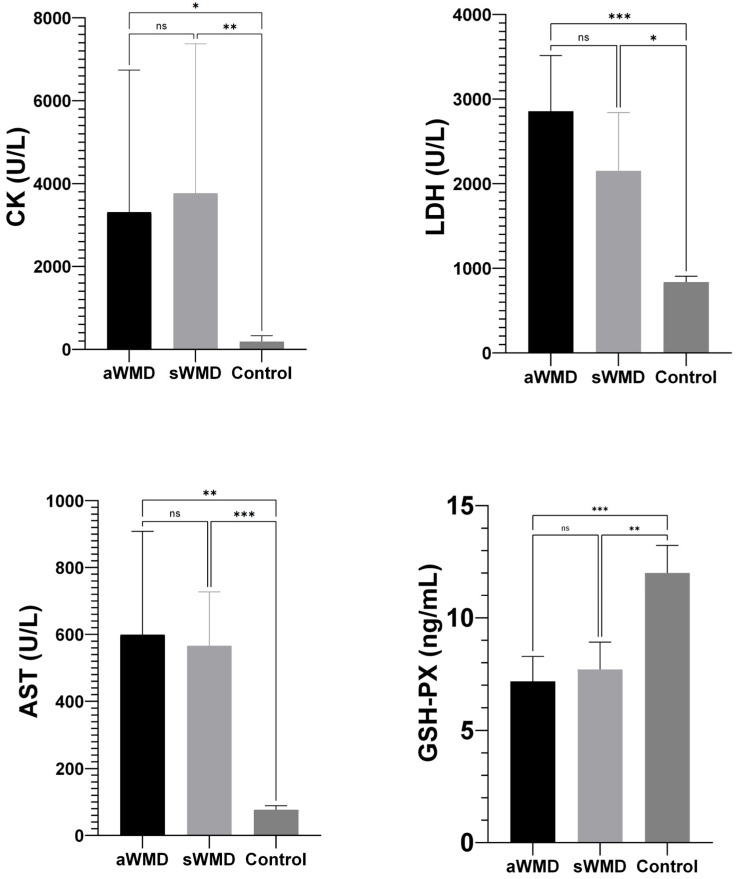
Comparison of biochemical parameters between case groups [aWMD: confirmed, severe white muscle disease (acute animals); sWMD: presumed white muscle disease (subacute animals)] and control groups. GSH-Px: glutathione peroxidase; CK: creatine kinase; LDH: lactate dehydrogenase; AST: aspartate aminotransferase. Data are expressed as mean ± 95% confidence interval (CI). aWMD: acute white muscle disease; sWMD: subacute white muscle disease. Control; healthy lambs. ns: non-significant. * *p* < 0.05, ** *p* < 0.01, *** *p* < 0.001.

The enzyme activities of CK (aWMD: 3312 U/L, sWMD: 3767 U/L), LDH (aWMD: 2859 U/L, sWMD: 2152 U/L), and AST (aWMD: 599.4 U/L, sWMD: 566 U/L) in the case group were significantly elevated compared to the control group (CK: 188 U/L, LDH: 838.7 U/L, AST: 76.67 U/L) with a significance level of *p* < 0.001. Conversely, the enzyme activity of GSH-Px (aWMD: 7.17 ng/mL, sWMD: 7.71 ng/mL) in the blood of lambs in the case group was markedly diminished in comparison to the control group (12.0 ng/mL) (*p* < 0.001, *p* = 0.001, respectively) (Figure 3).

### 3.6. Findings of Cardiac Biomarkers

The serum concentrations of NT-proBNP and Gal-3 in healthy lambs were 3.28 ± 0.71 ng/mL and 2.55 ± 0.52 ng/mL, respectively. Descriptive statistics for these parameters are presented in Table 2.

The average serum concentration of cTnI in lambs in aWMD (1.89 ng/mL) and sWMD (0.88 ng/mL) was different from each other, and both were significantly elevated compared to the control group (0.14 ng/mL) (*p* < 0.001). Similarly, serum CK-MB concentration in aWMD (204.3 U/L) exceeded that in sWMD (73.1 U/L) and the control group of healthy lambs (24.7 U/L) (*p* < 0.001) (Table 3). No significant difference was noted between groups regarding serum Gal-3 and NT-proBNP (*p* > 0.05) (Table 3).

In this study, when the threshold value for cTnI was taken as 0.18 ng/mL, sensitivity, specificity, and area under the ROC curve (AUC) were calculated as 100% (95% CI: 88.65–100), 95% (95% CI: 76.39–99.74), and 1.000, respectively. For CK-MB, sensitivity, specificity, and AUC values were determined as 88.24% (95% CI: 65.66–97.91), 100% (95% CI: 60.97–100), and 0.951, respectively. Sensitivity, specificity, and AUC values for AST, LDH, and CK variables are presented in Table 4.

A strong positive association was seen between cTnI and CK-MB (r = 0.819, *p* < 0.001) and LDH (r = 0.889, *p* < 0.001), while a moderate positive correlation was noted between CK (r = 0.629, *p* < 0.001) and AST (r = 0.732, *p* < 0.001). A strong positive association was observed between CK-MB and LDH (r = 0.803, *p* < 0.001), while a moderate negative correlation was identified with NT-proBNP (r = 0.551, *p* = 0.006). A significant negative connection was observed between Gal-3 and LDH (r = −0.421, *p* = 0.036), but a mild positive association was noted between NT-proBNP (r = 0.324, *p* = 0.022) (Table A1).

## 4. Discussion

This study examined blood levels of Gal-3, NT-proBNP, and other cardiac biomarkers in lambs diagnosed with WMD and healthy lambs; furthermore, clinical, macroscopic, and microscopic results were meticulously assessed in lambs within the acute WMD group. The data indicated that cTnI and CK-MB are relevant markers for assessing cardiac damage in both acute and subacute WMD; however, Gal-3 and NT-proBNP did not aid in diagnosis. Notably, NT-proBNP showed a tendency toward significance (*p* = 0.055) between the disease forms and the control group in lambs with WMD, suggesting it may have potential as a cardiac biomarker. Further studies in lambs and calves are needed to better evaluate the role of serum NT-proBNP in WMD and other cardiac conditions in farm animals.

In the present investigation, substantial abnormalities were seen in the cardiac and skeletal musculature of lambs in the aWMD group. This situation is consistent with the course of WMD. Prior research [24,27,28,29] has indicated that this condition, arising from Se and/or vitamin E insufficiency, has clinical manifestations including necrosis, calcification, and cellular infiltration in the musculature. In our investigation, extensive Zenker’s necrosis, dystrophic calcification, and indications of regeneration were noted in the myofibrils.

The current investigation determined the mean serum Gal-3 levels in healthy lambs to be 3.27 ng/mL (95% CI; 2.93–3.67). The data acquired from this study are significant as they serve as a crucial reference and comparative foundation for future comprehensive research. The average blood Gal-3 content in felines is documented as 0.27 ± 0.15 pg/mL [23]. In canines, Gal-3 levels exhibit considerable variability; specifically, one study indicated a serum Gal-3 concentration of 0.64 ± 0.15 ng/mL in healthy dogs [22], whilst another study revealed a value of 3.90 ± 1.65 ng/mL [30]. The reference ranges for Gal-3 serum levels in horses remain inadequately defined, and there is a paucity of studies addressing this topic in the literature [31]. On the other hand, there are no data on serum levels of Gal-3 in ruminants (cattle, sheep, goats). These differences observed in serum Gal-3 concentrations in healthy animal species can be attributed to factors including physiological changes between species, age, sex, and environmental influences.

In our investigation, Gal-3 levels exhibited no statistically significant difference between the case and control groups. WMD is an acute condition marked by abrupt muscle necrosis and disintegration [32]. Nevertheless, elevated Gal-3 levels are more evident in prolonged disorders characterized by chronic inflammation [17] and fibrotic responses [23,33,34]. A study on cats with hypertrophic cardiomyopathy indicated that elevated serum Gal-3 levels correlated with disease severity and significantly contributed to the pathophysiology by mediating inflammation and fibrosis [23]. The absence of a notable increase in Gal-3 levels throughout the acute and subacute phases of WMD in our study can be attributed to the acute characteristics of WMD and the lack of established fibrotic processes.

NT-proBNP is extensively utilized with additional tests for diagnosing cardiac dysfunction or heart failure in humans and several animal species [9,13,14,15]. NT-proBNP typically elevates in response to ventricular wall stress [9,13,14,15]. In a sheep model of experimental myocardial infarction, it was observed that plasma concentrations of brain natriuretic peptide (BNP) and NT-proBNP were elevated in comparison to the control group following chronic volume overload induced by intravenous saline [15]. The current study does not provide evidence supporting a relationship between cardiac dysfunction caused by WMD and NT-proBNP. This can be explained by the fact that WMD develops a picture of heart failure due to the loss of the ability of the heart muscle to contract and relax due to degeneration and necrosis of the heart muscle fibers and myofibrils, thus decreasing the pumping power and efficiency of the heart, rather than myocardial strain (excessive strain or stress of the heart muscle) and ischemia.

Cardiac troponin I (cTnI) is a protein exclusive to cardiac myocytes and serves as one of the most sensitive and specific cardiac biomarkers released into the bloodstream following cardiomyocyte injury [27,28]. The typical cTnI concentration in the blood serum of healthy lambs ranges from 0 to 0.21 ng/mL [35]. In lambs with WMD, the serum levels of cTnI may rise to 0.2–3.0 ng/mL or more, contingent upon the extent of myocardial injury [32]. In accordance with the findings of previous studies [25,27,32,36], serum cTnI levels were considerably elevated in lambs with WMD relative to healthy controls. The elevation of cTnI observed in WMD signifies direct impairment and cellular degeneration of cardiac muscle tissue [37,38]. Vitamin E and selenium are fundamental elements of cellular antioxidant defense; their deficiencies elevate oxidative stress, inflict structural damage to cell membranes and proteins, leading to muscle degeneration and compromised cellular integrity [4,39].

CK-MB is mostly released into the bloodstream as a result of myocardial cell injury [35,40]. Deficiencies in vitamin E and/or selenium induce oxidative damage in muscle cells, resulting in cell membrane breakdown and the release of intracellular components, including CK-MB, into the bloodstream [41]. The current investigation revealed that serum CK-MB levels were markedly elevated in lambs with WMD compared to healthy controls, corroborating the results of other studies [27,32,37].

In detecting myocardial damage, cTnI is superior to CK, CK-MB, AST and LDH [42]. The current investigation revealed that cardiac markers cTnI and CK-MB were considerably elevated in lambs with WMD compared to healthy counterparts, with cTnI demonstrating exceptional diagnostic efficacy for heart injury, exhibiting 100% sensitivity and 95% specificity. While cTnI serves as a very sensitive and specific marker for cardiac muscle injury, it is inadequate on its own to ascertain the etiology of the damage [27,43,44]. CK-MB levels may also be elevated in individuals with myopathy, neuropathy, skeletal muscle injury, or renal failure, independent of cardiac disease [45]. Elevations in serum cTnI and CK-MB levels may occur in viral myocarditis associated with foot and mouth disease [27,43,44,46], babesiosis [47], acute ruminal lactic acidosis [48], pregnancy toxaemia [49], chronic mitral valve disease [50], canine parvoviral enteritis [51], traumatic reticuloperitonitis [52], canine distemper [51], and dilated cardiomyopathy [51]. Human studies have benefited from extensive and rigorous evaluations on this issue due to the large number of instances and the sophisticated and prevalent application of laboratory testing. This is a methodological constraint in the present investigation, hence restricting the applicability of these biomarkers in the diagnosis of WMD.

Biochemical tests indicated that the activities of CK, LDH, and AST enzymes, which serve as markers of muscle injury, were significantly elevated in lambs with WMD. Deficiency of vitamin E and/or selenium induces oxidative stress in muscle cells, compromising cell membrane integrity and muscle tissue, which subsequently elevates serum enzyme concentrations [2,25,27,53,54]. The elevation of CK, AST, and LDH levels further confirmed that these enzymes are reliable indicators in diagnosing muscle injury resulting from WMD. ROC analysis indicated that CK, LDH, and AST are effective biomarkers for diagnosing WMD, exhibiting good sensitivity and specificity.

CK-MB levels can increase not only due to myocardial injury but also as a result of skeletal muscle degeneration [32,35,37,54]. Therefore, the cardiac specificity of CK-MB is not well established, particularly in ruminant species. Studies have shown that, unlike in humans [41], CK-MB levels in animals such as calves [55] and pigs [56] do not reliably reflect myocardial injury. For example, in neonatal calves exposed to endotoxemia, serum CK-MB levels remained low despite myocardial stress, whereas cardiac troponin I increased significantly [55]. Similarly, in pig models, both myocardial and skeletal muscle damage led to an elevation in total CK, but not in CK-MB, indicating limited cardiac specificity [56]. However, in the present study, a significant correlation was observed between CK-MB levels and troponin, a cardiac-specific biomarker, in lambs, suggesting that the increase in CK-MB may be of cardiac origin. This finding supports the interpretation of increased CK-MB as a potential indicator of myocardial damage.

The levels of vitamin E and selenium were markedly diminished in the case groups compared to the control group, as anticipated. This outcome reaffirms the significance of vitamin E and selenium insufficiency, which are primary etiological components of WMD, in diagnosis [4,32]. The observation of clinical improvement post-treatment in lambs within the sWMD group demonstrates the efficacy of this element and vitamin in adjunctive therapy. It is well documented that certain regions of Türkiye, including parts of central Anatolia are prone to selenium deficiency, which significantly reduces the selenium content of locally produced forage and grains [3,4,24,37]. Therefore, it is likely that the lambs originated from a naturally selenium-deficient environment, contributing to the observed deficiency. In this study, the lambs were fed a uniform dry ration with no access to green forage, suggesting that the vitamin E deficiency was more likely due to insufficient dietary intake of natural antioxidants rather than excessive polyunsaturated fatty acids intake.

One of the limitations of this study is that the definitive diagnosis of the lambs included in the sWMD group was not confirmed by gold-standard methods such as histopathological examination. The diagnosis for this group was made presumptively based on clinical signs, serum selenium and vitamin E levels, and laboratory findings such as muscle enzyme activities. Therefore, the lack of diagnostic certainty for sWMD is an important limitation that should be carefully considered when interpreting the findings and generalizing the results.

## 5. Conclusions

In conclusion, the findings obtained from this study indicate that cTnI and CK-MB may be useful in determining cardiac dysfunction due to white muscle disease, whereas Gal-3 and NT-proBNP levels do not have a significant diagnostic value. However, considering the possibility that Gal-3 may increase significantly in more chronic stages of white muscle disease or the presence of fibrotic heart lesions, further research on this subject is recommended.

## Figures and Tables

**Table 1:** Comparison of body temperature, respiratory rate, and heart rate between groups.

Parameters	aWMD (*n* = 10)	sWMD (*n* = 20)	Control (*n* = 20)	*p* Values
T (°C)	39.7 ± 1.01 ^a^	38.3 ± 0.32 ^b^	39.0 ± 0.28 ^ab^	<0.001
HR (beats/min)	235.0 ± 29.50 ^a^	140.2 ± 42.75 ^b^	125.2 ± 19.76 ^a^	<0.001
RR (breaths/min)	95.40 ± 52.41 ^a^	35.33 ± 8.17 ^ab^	35.4 ± 3.04 ^b^	0.01

Data were expressed as mean ± standard deviation. aWMD: confirmed, severe white muscle disease (acute animals) lambs; sWMD: presumed white muscle disease (subacute animals); Control: healthy lambs; HR: heart rate; RR: respiratory rate; T: temperature. Values in the same row with different superscript letters (a, b) are significantly different from each other (*p* < 0.05).

**Table 2:** Serum NT-proBNP and Gal-3 concentrations of healthy lambs (*n* = 20).

Parameters	Mean	Lower and Upper 95% CI of Mean	Median	25th–75th Percentile	Min–Max
Gal-3 (ng/mL)	3.28 ± 0.71	2.94–3.61	3.28	2.79–3.60	2.14–5.22
NT-proBNP (ng/mL)	2.55 ± 0.52	2.31–2.79	2.61	2.18–3.00	1.63–3.33

Gal-3: Galectin-3; NT-proBNP: N-terminal pro B-type natriuretic peptide; Min: minimum; Max: Maximum; CI: confidence interval.

**Table 3:** Comparison of cardiac biomarkers between groups.

Parameters	aWMD (*n* = 10)	sWMD (*n* = 20)	Control (*n* = 20)	*p* Values
cTnI (ng/mL)	1.89 ± 0.05 ^a^	0.80 ± 0.04 ^b^	0.14 ± 0.02 ^c^	<0.001
Gal-3 (ng/mL)	2.99 ± 0.44	3.07 ± 0.42	3.28 ± 0.71	0.350
NT-proBNP (ng/mL)	2.15 ± 0.32	2.64 ± 0.55	2.55 ± 0.52	0.055
CK-MB (U/L)	204.30 ± 85.20 ^a^	73.14 ± 29.84 ^b^	24.67 ± 8.96 ^b^	<0.001

Data were expressed as mean ± standard deviation or median (25th–75th percentile). aWMD: confirmed, severe white muscle disease (acute animals); sWMD: presumed white muscle disease (subacute animals). Control: healthy lambs. cTnI: cardiac troponin I; CK-MB: creatine kinase myocardial band; Gal-3: galectin-3; NT-proBNP: N-terminal B type natriuretic peptide. Values in the same row with different superscript letters (a, b, c) are significantly different from each other (*p* < 0.05).

**Table 4:** Sensitivity, specificity and area under the ROC curve (AUC) values of the threshold values for AST, LDH, CK, cTnI and CK-MB.

Marker	AUC	95% Cl		SE	*p* Values
cTnI (ng/mL)	1.000	1.000 to 1.000		0.000	<0.001
CK-MB (U/L)	0.951	0.867 to 1.000		0.043	0.001
AST (U/L)	0.994	0.975 to 1.000		0.010	<0.001
LDH (U/L)	1.000	1.000 to 1.000		0.000	<0.001
CK (U/L)	0.920	0.815 to 0.100		0.053	<0.001
		**Sensitivity analysis**			
**Marker**	**Cut-off value**	**Sensitivity** **% (95% CI)**	**Specificity** **% (95% CI)**	**Max. Youden’s** **Index**	
cTnI (ng/mL)	0.18	100 (88.65–100.0)	95 (76.39–99.74)	0.95	
CK-MB (U/L)	55.0	88.24 (65.66–97.91)	100.0 (60.97–100)	0.88	
AST (U/L)	97.0	100 (78.47–100.0)	91.67 (64.61–99.57)	0.92	
LDH (U/L)	900	100 (83.18–100)	83.33 (43.65–99.15)	0.83	
CK (U/L)	485	72.22 (49.13–57.50)	88.89 (56.50–99.43)	0.61	

AUC: Area under the ROC curve; Cl: confidence interval; SE: standard error; cTnI: cardiac troponin I; CK-MB: creatine kinase myocardial band; AST: aspartate aminotransferase; CK: creatine kinase; LDH: lactate dehydrogenase.

## Data Availability

The data supporting this study’s findings are available from the corresponding author upon reasonable request.

## Abbreviations

The following abbreviations are used in this manuscript:

WMD	White muscle disease
Gal-3	Galectin-3
cTnI	Cardiac troponin I
NT-proBNP	N-terminal pro-B type natriuretic peptide
CK-MB	Creatine kinase myocardial band
aWMD	Confirmed cases of severe acute white muscle disease in lambs
sWMD	Presumed cases of subacute white muscle disease in lambs
Se	Selenium

## Appendix A

**Table A1:** Spearman’s rho correlation results.

	cTnI	CK-MB	Gal-3	NT-proBNP	CK	LDH	AST
cTnI	1000						
CK-MB	0.819 **	1000					
Gal-3	−0.180	−0.383	1.000				
NT-proBNP	−0.154	−0.551 **	0.324 *	1.000			
CK	0.629 **	0.420 *	−0.003	−0.205	1.000		
LDH	0.889 *	0.803 **	−0.421 *	−0.364	0.567 **	1.000	
AST	0.732 **	0.489 *	−0.033	0.191	0.611 **	0.562 **	1.000

cTnI: cardiac troponin I; CK-MB: creatine kinase myocardial band; Gal-3: Galectin-3; NT-proBNP: N-terminal B type natriuretic peptide; AST: aspartate aminotransferase; CK: creatine kinase; LDH: lactate dehydrogenase. * Correlation is significant at the 0.05 level (2-tailed). ** Correlation is significant at the 0.01 level (2-tailed).
